# Clinical value of SPECT/CT for evaluation of patients with painful knees after total knee arthroplasty- a new dimension of diagnostics?

**DOI:** 10.1186/1471-2474-12-36

**Published:** 2011-02-04

**Authors:** Michael T Hirschmann, Praveen Konala, Farhad Iranpour, Anna Kerner, Helmut Rasch, Niklaus F Friederich

**Affiliations:** 1 Department of Orthopaedic Surgery and Traumatology, Kantonsspital Bruderholz, CH-4101 Bruderholz, Switzerland; 2 Musculoskelettal Surgery Department, Imperial College, London, UK; 3 Institute of Radiology and Nuclear Medicine, Kantonsspital Bruderholz, CH-4101 Bruderholz, Switzerland

## Abstract

**Background:**

The purpose of our study was to evaluate the clinical value of hybrid SPECT/CT for the assessment of patients with painful total knee arthroplasty (TKA).

**Methods:**

Twenty-three painful knees in patients following primary TKA were assessed using Tc-99m-HDP-SPECT/CT. Rotational, sagittal and coronal position of the TKA was assessed on 3D-CT reconstructions. The level of the SPECT-tracer uptake (0-10) and its anatomical distribution was mapped using a validated localization scheme. Univariate analysis (Wilcoxon-Mann-Whitney, Spearmean`s-rho test, p < 0.05) was performed to identify any correlations between component position, tracer uptake and diagnosis.

**Results:**

SPECT/CT imaging changed the suspected diagnosis and the proposed treatment in 19/23 (83%) knees. Progression of patellofemoral OA (n = 11), loosening of the tibial (n = 3) and loosening of the femoral component (n = 2) were identified as the leading causes of pain after TKA.

Patients with externally rotated tibial trays showed higher tracer uptake in the medial patellar facet (p = 0.049) and in the femur (p = 0.051). Patients with knee pain due to patellofemoral OA showed significantly higher tracer uptake in the patella than others (p < 0.001).

**Conclusions:**

SPECT/CT was very helpful in establishing the diagnosis and guiding subsequent management in patients with painful knees after TKA, particularly in patients with patellofemoral problems and malpositioned or loose TKA.

## Background

Total knee arthroplasty (TKA) is the treatment of choice for patients with disabling primary osteoarthritis (OA) of the knee joint. Although TKA is a very successful surgical procedure in patients with OA and it generally leads to satisfactory long-term results, failure does occur in a considerable number of patients resulting in persistent or recurrent knee pain [1-4]. The most common causes are considered to be infection, loosening, instability, prosthetic malposition, arthrofibrosis and patellofemoral disorders [1-4]. Clinically it can be difficult to differentiate between causes which necessitate surgical treatment from those which could be treated non-surgically [1-3]. Hence, identifying the underlying cause of the pain is of paramount importance for guidance of optimal patient management. To date no optimal `single-stage` sensitive and specific diagnostic imaging modality, which integrates mechanical and metabolic data has been reported for this group of patients [1-3,5,6].

Radiographs are considered to be the primary standard imaging technique in patients with knee pain after TKA [1-3]. However, these are only helpful in detecting gross prosthetic malposition, radiolucencies and fractures. Plain radiographs are less sensitive in detecting more common but subtle abnormalities such as early loosening or minor implant malposition [1-3]. Radiographs are also subject to measurement inaccuracy due to variability in reproducible patient positioning [5-7].

Computer tomography (CT) has its value in identifying TKA malposition and may reveal the extent and size of periprosthetic lucencies not apparent on plain radiographs [8,9]. Although bone scans or single emission computerized tomography (SPECT) give important information on the osseous metabolism and joint homeostasis [10,11] their clinical value is limited due to the poor accuracy in localizing the increased tracer uptake [12]. Hybrid SPECT/CT which combines the strengths of SPECT and CT may be useful in patients with knee pain after TKA, particularly when other radiographic imaging provides insufficient, ambigous or non-specific information [5,6].

The primary purpose of this study was to evaluate the clinical value of SPECT/CT in patients with knee pain after primary TKA. The hypothesis was that the use of SPECT/CT has a substantial clinical impact in terms of establishment of diagnosis and guidance of further management in these patients.

## Methods

### Patients

A total of 23 consecutive patients who have previously undergone primary TKA and complained about postoperative knee pain were prospectively collected and investigated. The patients were all recruited during a 6 months period at a university affiliated hospital specialized in knee surgery. Patients who had undergone a revision surgery previously were excluded. There were no other exclusion criteria.

All patients (mean age 69 ± 13 years, range 38-88 years) underwent clinical and radiological examination including standardized radiographs (anterior-posterior and lateral weight bearing, patellar skyline view) and Tc-99m-HDP-SPECT/CT. The mean time from primary TKA to the date of SPECT/CT imaging was 60 ± 45 months.

Age, gender, side, time from primary TKA, type of primary TKA, diagnosis before and after SPECT/CT, final diagnosis and treatment was noted. The final diagnosis was based on intraoperative (when revised n = 8) or microbiological and histological examinations (n = 8), clinical and radiological findings (n = 15).

Data was analysed to determine whether SPECT/CT had changed the diagnosis and/or subsequent treatment. The study was approved by our Institutional Review Board.

### Radiological Imaging

Tc-99m-HDP-SPECT/CT was performed using a hybrid system (Symbia T16, Siemens, Erlangen, Germany) which consists of a pair of low energy, high-resolution collimators and a dual-head gamma camera and an integrated 16 × 0.75-mm slice-thickness CT. All patients received a commercial 700 MBq Tc-99 m HDP injection (CIS Bio International Sur Yvette, France). Planar scintigraphic images were taken in the perfusion phase (immediately after injection), the soft tissue phase (1 to 5 minutes after injection) and the delayed metabolic phase (2 hours after injection). SPECT/CT was performed with a matrix size of 128 × 128, an angle step of 32, and a time per frame of 25 seconds two hours after injection.

**Figure 1:**
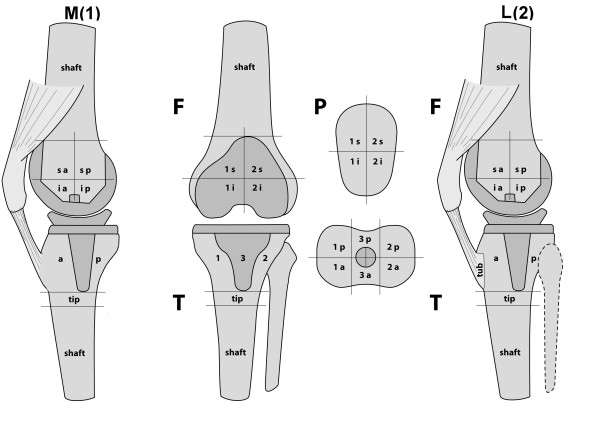
**The previously published and used SPECT/CT classification system**. (Reprint permission from Journal KSSTA, Springer).

Data were processed by interactive reconstruction on a computer workstation (Syngo, Siemens, Erlangen, Germany). Images were displayed in orthogonal axial, coronal and sagittal planes and interpreted by one specialized nuclear radiologist. The tracer activity on SPECT/CT was noted using a system based colour-coded grading scale (0-10). The localization of the tracer activity was recorded on a validated standardized localization scheme (Figure 1). The intra- and inter-observer reliability of the measurements have been described previously. ICC values were >0.9, reflecting a very reliable methodology [6]. The highest activity grading for each area of the localization scheme and whether the area of tracer activity extended to the bone prosthesis interface was noted.

The rotational (internal-external rotation), sagittal (flexion-extension, anterior-posterior slope) and coronal (varus-valgus) alignment of the prostheses were assessed on 3D reconstructed CT images using a customized software. The exact measurement procedure has been previously described [6]. As the head of the femur and the mid-ankle was not available in these CT images, the femoral and tibial anatomical axis were determined by modelling cylinder of best fit to the femoral and tibial shafts. The axis of the cylinder was assumed to represent the anatomical axis.

### Statistical Analysis

All data were analyzed by an independent statistician using SPSS version 16.0 (SPSS, Chicago, U.S.A.). Continuous variables were described using means and standard deviations or medians and ranges. Categorical variables were tabulated with absolute and relative frequencies. Univariate analysis (Wilcoxon-Mann-Whitney, Spearmean`s-rho test, p < 0.05) was performed to identify any correlations between component position, tracer uptake and diagnosis. For all analysis, p < 0.05 was considered statistically significant.

## Results

The patients` demographics, follow-up from primary TKA, type of primary TKA, the diagnosis before and after SPECT/CT imaging (final diagnosis) and subsequent treatment are presented in table 1.

**Table 1:** Patients` demographics, time from primary TKA, suspected diagnosis before SPECT/CT, final diagnosis and performed treatment

**No**.	Initials	Sex	Age (yrs)	Time from primary TKA (mths)	Side	Type of primary prosthesis	Suspected diagnosis before SPECT/CT	Final diagnosis after SPECT/CT	Final treatment
1	R.R.	m	50	56	R	Natural Knee^®^, Zimmer (tibial cemented, femoral uncemented)	Loosening of tibial component	Patellofemoral OA	Patellar resurfacing

2	H.U.	f	73	29	R	Natural Knee^®^, Zimmer (tibial cemented, femoral uncemented)	Loosening of tibial and/or femoral component	Patellofemoral OA	Non-surgically

3	S.A.	f	85	59	R	BalanSys^®^, Mathys (tibial cemented, femoral uncemented)	Patellofemoral hyperpression	Patellofemoral hyperpression due to flexion of femoral component; external malrotation >10°of tibial component	Non-surgically

4	F.L.	f	79	36	L	Triathlon^®^, Stryker (tibial cemented, femoral uncemented)	Loosening of tibial and/or femoral component	Insertion tendinopathy of iliotibial tract due to external malrotation of tibial component >15°; posterior tibial slope <3°	Non-surgically

5	P.M.	f	55	68	L	BalanSys^®^, Mathys (tibial cemented, femoral uncemented)	Infection	Patellofemoral OA	Patellar resurfacing

6	L.S.	f	54	20	L	Triathlon^®^, Stryker (tibial cemented, femoral uncemented)	Infection, malrotation of tibial component	Patellofemoral OA; posterior, tibial slope <3°,	Patellar resurfacing

7	K.G.	f	77	116	L	LCS^®^, Depuy (tibial cemented, femoral uncemented)	Loosening of tibial component	Loosening and external malrotation of tibial component >15°, posterior tibial slope >15°	Revision tibial component

8	M.D.	f	65	32	R	LCS^®^, Depuy (tibial cemented, femoral uncemented)	Loosening of tibial component	Patellofemoral OA	Patellar resurfacing

9	H.R.	m	67	48	L	BalanSys^®^, Mathys (tibial cemented, femoral uncemented)	Loosening of femoral component	Loosening of femoral component	Revision planned

10	A.C.	f	38	36	R	LCS^®^, Depuy (tibial cemented, femoral uncemented)	Loosening of femoral and/or tibial component	Incipient loosening of tibial component and external malrotation >10°	Non-surgically

11	S.C.	f	88	212	R	LCS^®^, Depuy (tibial cemented, femoral uncemented)	Breakage of polyethylene inlay, loosening of tibial component	Breakage of polyethylene inlay, posterior tibial slope 14°	Change of inlay

12	R.C.	f	52	56	R	TC Plus™, Smith&Nephew (tibial cemented, femoral uncemented)	Loosening of tibial and/or femoral component after periprosthetic fissure	No evidence for loosening; consolidation of periprosthetic fracture, patellofemoral OA	Non-surgically

13	W.M.	m	74	34	R	LCS^®^, Depuy (tibial cemented, femoral uncemented)	Loosening of tibial and/or femoral component	External malrotation of tibial component >15°	Revision recommended, treated non-surgically due to comorbidities

14	K.A.	f	77	48	L	Natural Knee^®^, Zimmer (tibial cemented, femoral uncemented)	Loosening of tibial and/or femoral component; low grade infection	No evidence for loosening. No malrotation.	Patient underwent physiotherapy; to date asymptomatic.

15	S.H	m	79	18	L	Triathlon^®^, Stryker (tibial cemented, femoral uncemented)	Loosening of tibial and/or femoral component	Patellofemoral OA	Patellar resurfacing

16	S.H.	m	63	60	L	TC Plus™, Smith&Nephew (tibial cemented, femoral uncemented)	Loosening of tibial and/or femoral component	Loosening of tibial component	Revision tibial component

17	S.H.	m	63	60	R	BalanSys^®^, Mathys (tibial cemented, femoral uncemented)	Loosening of tibial and/or femoral component	Patellofemoral OA, external malrotation >10° of tibial component	Non-surgically

18	B.W.	m	72	48	L	Triathlon^®^, Stryker (tibial cemented, femoral uncemented)	Loosening of tibial and/or femoral component	No evidence for loosening. No malrotation	Patient underwent physiotherapy; to date asymptomatic.

19	M.D.	f	60	9	R	Triathlon^®^, Stryker (tibial cemented, femoral uncemented)	Loosening of tibial component, patellar OA	Patellofemoral OA, posterior tibial slope <3°	Planned Patellar resurfacing

20	F.L.	f	78	9	R	Triathlon^®^, Stryker (tibial cemented, femoral uncemented)	Early loosening of tibial component, patellar OA	Patellofemoral OA, external malrotation >15° of tibial component	Planned Patellar resurfacing

21	L.R.	f	61	18	R	Natural Knee^®^, Zimmer (tibial cemented, femoral uncemented)	Oversized femoral and tibial component	Persistent synovitis due to oversized femoral and tibial component, tibial internal malrotation >10°	Femoral and tibial component revision

22	K.L.	m	80	104	L	LCS^®^, Depuy (tibial cemented, femoral uncemented)	Loosening of femoral and/or tibial component	Loosening of femoral component and tibial external malrotation >15°	Revision recommended, treated non-surgically due to comorbidities

23	L.J.	f	77	108	R	LCS^®^, Depuy (tibial cemented, femoral uncemented)	Loosening of femoral and/or tibial component	No evidence for loosening. No malrotation	Patient underwent physiotherapy; to date asymptomatic.

**Figure 2:**
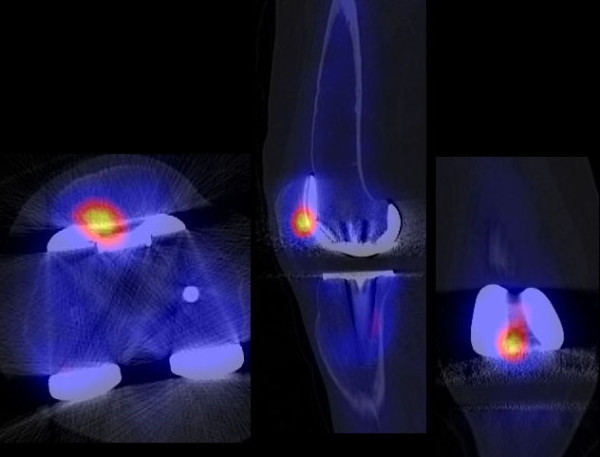
**SPECT/CT imaging of a patient with patellofemoral problems as cause of painful total knee arthroplasty**.

In 11/23 patients progression of patellofemoral OA was identified as the cause of knee pain and seven of these 11 cases have been revised or scheduled for revision (Figure 2). Three patients with symptomatic patellofemoral OA being high risk surgical patients were reluctant to undergo revision surgery. One patient in this group had a periprosthetic fracture and a decision was made to postpone surgery until the fracture had healed. Six patients with symptomatic patellofemoral OA showed tibial component malrotation.

**Figure 3:**
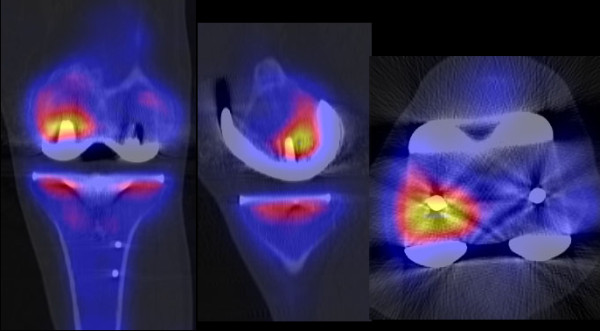
**SPECT/CT imaging of a patient with mechanical loosening of femoral component as cause of painful total knee arthroplasty**. (Reprint permission from Journal KSSTA, Springer).

**Figure 4:**
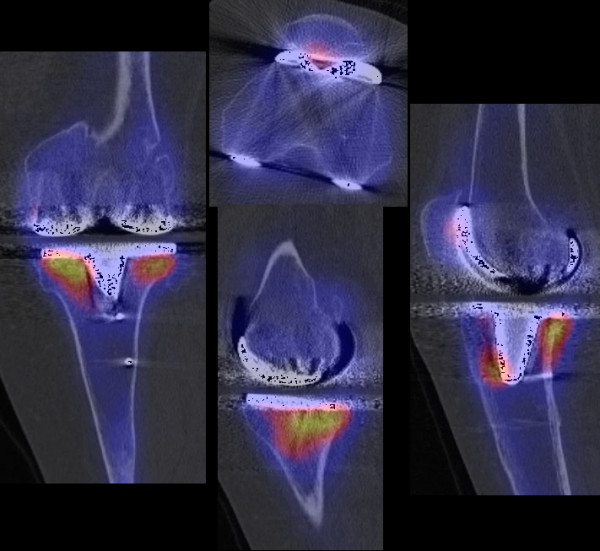
**SPECT/CT imaging of a patient with mechanical loosening of tibial component as cause of painful total knee arthroplasty**.

Other leading causes of knee pain noted in this study were tibial (n = 3) and femoral component loosening (n = 2). Loosening of the femoral component (n = 2) was associated with external malrotation of the tibial component (>10°) in 1 patient (Figure 3). Loosening of the tibial component (n = 3) was associated with external malrotation of tibial component (>10°) in 2 patients; one of these also showed a posterior tibial slope >15° (Figure 4). In one patient the femoral and tibial component was oversized and internally malrotated causing persistent synovitis.

A clinically relevant change in diagnosis and proposed treatment before and after SPECT/CT was observed in 19/23 cases. In all patients (n = 8) being revised to date the diagnosis after SPECT/CT imaging matched with the intraoperative findings.

The Tc-99m-HDP tracer activity and allocation to each anatomical area are presented in table 2.

**Table 2:** Absolute Tc-99m-HDP SPECT/CT tracer activity localized with the "Bruderholz" scheme ("c" indicates contact of uptake area to prosthesis interface)

**No**.	Tibia									Femur								
	**1**		**2**		**3**		**Others**			**1**				**2**				**other**

	**a**	**p**	**a**	**p**	**a**	**p**	**tip**	**Tibtub**	**shaft**	**sa**	**ia**	**sp**	**ip**	**sa**	**ia**	**sp**	**ip**	**shaft**

**1**	5	5c	4	5c	0	0	0	0	1	0	0	0	7	5c	0	0	7	1

**2**	0	5c	0	5	0	6	0	0	2	0	3	0	4	0	3	0	7c	2

**3**	0	6	10c	8	0	0	0	6	1	0	0	0	5c	0	0	0	7c	1

**4**	10c	10c	4	5	6	0	0	6	2	0	7c	4	7c	0	0	3	6c	2

**5**.	0	3	6	0	0	0	0	0	3	0	0	0	0	0	0	0	0	2

**6**	6	7	5	7	0	0	0	4	2	7c	5	5	9c	5	6	5	9	2

**7**	6	10c	7c	7c	0	10c	10c	10	2	4	4	0	4c	4	3	0	4c	1

**8**	7c	8c	7c	8c	0	5	5	6	2	5	7	0	7	6	7	0	7	1

**9**	6	7	6	8	5	5	0	7	1	7	6	0	10c	0	0	0	7c	2

**10**	6	5	5	9c	10	10c	10c	0	2	4	3	0	5c	4	5c	0	6c	1

**11**	4c	4c	5c	8c	0	0	0	0	1	4	3	0	7c	5	5c	0	10c	1

**12**	5c	5c	4c	6c	0	0	0	6	1	4	4	0	6c	6	3	0	4c	1

**13**	0	0	0	0	0	3	3	3	1	0	0	0	3c	0	0	0	3c	1

**14**	9c	9c	7c	8c	4c	7c	3c	4	3	3	3	5	7c	4	10c	5	6	3

**15**	4c	4c	5c	5c	4	2	1	4	1	3c	4	4c	6	5c	5c	7c	10	3

**16**	7	7	7c	6c	3c	3c	2	3	2	2	3c	3	4c	4c	4c	4c	5c	2

**17**	3	5c	3c	3c	3c	2c	5c	3	3	1	3c	3	3c	1	2c	2	4c	1

**18**	3	2	3	3	2c	1	2	2	1	2	2c	4c	3	2	3	3c	3	2

**19**	4	4	3c	3c	4c	3c	5c	4	1	4c	4c	4c	5c	4c	5c	9c	10c	1

**20**	4	7c	7c	4c	3c	2c	1	4	1	3c	3c	4c	4c	4c	4c	4c	6c	1

**21**	4	4	3	5c	1	1	1	1	1	2	3c	7c	7c	2	3c	2c	5c	1

**22**	4	4	3	3	0	4	4	2	5	4	5c	3	5c	3	3	2	0	1

**23**	4	6	3	4	0	3	3	0	0	3	4	0	4	3	3	0	3	1

**Median**	4	5	5	5	0	2	1	3	2	3	3	0	5	4	3	0	6	1

**Min**	0	0	0	0	0	0	0	0	1	0	0	0	0	0	0	0	0	1

**Max**	10	10	10	9	10	10	10	10	3	7	7	7	10	6	10	9	10	3

The CT measurements of femoral and tibial component position are shown in table 3.

**Table 3:** The rotational 3D-CT measurements of patients after total knee arthroplasty

**No**.	3D-CT measurements					
	**Femoral**			**Tibial**		

	**Varus (+)/Valgus (-)**	**Flexion(+)/Extension (-)**	**Internal (+)/External rotation (-)**	**Varus (+)/Valgus (-)**	**Posterior slope**	**Internal (+)/External rotation (-)**

1.	-5	-5	0	5	10	2

2	-7	1	6	8	2	-8

3	4	1	2	0	10	-14

4	-8	3	5	8	2	-20

5	-2	3	4	1	8	-5

6.	-2	0	1	3	1	9

7	-9	0	4	3	19	-29

8	-4	5	2	4	6	-1

9.	-4	-4	6	4	8	2

10	-5	6	5	6	10	-14

11	5	9	4	2	14	-9

12	-12	0	0	3	5	-8

13	-9	3	9	5	7	-17

14	-8	-2	3	1	8	5

15	-8	1	0	4	4	-4

16	-8	-2	5	4	10	1

17	-5	5	-1	4	9	-15

18	-6	-1	7	-1	10	1

19	1	1	0	-1	1	-5

20	-8	9	3	2	5	-20

21	-9	6	4	4	9	11

22	2	5	-5	5	4	-16

23	-5	11	-5	5	9	-17

Patients with externally malrotated tibial tray showed significantly higher tracer activity in the medial patellar facet (p = 0.049) and by trend in the femur (p = 0.051).

Posterior tibial slope <3° or >10° was associated with increased femoral tracer uptake (p = 0.040). Patients with patellofemoral OA as leading cause for their knee pain showed significantly (p < 0.0001) higher tracer uptake in the patella than others (table 4).

**Table 4:** Tc-99m-HDP tracer activity within the patella in patients with patellofemoral OA as cause for revision versus others

**No**.	Initials	1i	1s	2i	2s	Median value	Highest Value medial	Highest value lateral
**Patients with patellofemoral OA a s cause of knee pain**								

**1**	R.R.	6	8	8	10	8	8	10

**2**	H.U.	7	9	7	10	8	9	10

**3**	S.A.	6	4	9	5	5.5	6	9

**5**	P.M.	10	9	10	9	9.5	10	10

**6**	L.S.	7	8	8	10	8	8	10

**8**	M.D.	6	6	7	6	6	6	7

**12**	R.C.	10	9	10	9	9.5	10	10

**15**	S.H	10	9	7	7	8	10	7

**17**	S.H.	7	6	7	10	7	7	10

**19**	M.D.	6	8	7	6	6.5	8	7

**20**	F.L.	10	10	10	10	10	10	10

**Patient with other causes for knee pain**								

**4**	F.L.	4	4	6	6	5	4	6

**7**	K.G.	6	5	6	6	6	5	6

**9**	H.R.	5	4	4	3	4	5	4

**10**	A.C.	7	6	8	8	7.5	6	8

**11**	S.C.	8	8	6	6	7	8	6

**13**	W.M.	3	3	3	4	3	4	4

**14**	K.A.	8	7	8	5	7.5	8	8

**16**	S.H.	6	4	6	4	5	6	6

**18**	B.W.	2	2	3	2	2	2	3

**21**	L.R.	5	3	8	5	5	5	8

**22**	K.L.	3	4	4	3	3.5	4	4

**23**	L.J.	4	3	3	3	3	4	3

Spearman`s rho correlation between groups .713 (p = 0.000) .697 (p = 0.000) .743 (p = 0.000)

Patients with loosening of the femoral component showed significantly higher tracer uptake in the tibia, which also extended to the prosthetic interface (p = 0.023). The femoral uptake was also higher, but did not reach statistical significance (p = 0.135). Patients with loosening of the tibial component showed higher tracer uptake in the tibia, but the difference was not statistically significant (p = 0.216).

## Discussion

Integrated hybrid SPECT/CT is increasingly considered to be a promising new diagnostic imaging modality for orthopaedic patients [5,6,13]. To date SPECT/CT has not been frequently used among the orthopaedic fraternity [5,6,13,14].

The primary purpose of this study was to evaluate the clinical value of Tc-99m-HDP-SPECT/CT in patients with knee pain after primary TKA.

The most important findings of our study are threefold:

Firstly, SPECT/CT imaging significantly changed the diagnosis and treatment proposed, independently of previous intention to revise or treat the patient non-surgically. In addition, the established diagnosis after SPECT/CT imaging was confirmed intraoperatively in all patients who have undergone revision surgery.

SPECT/CT proved to be particularly helpful in identifying patellofemoral OA, which was responsible for knee pain in nearly half of our patients. Patellofemoral problems such as progression of OA, instability and maltracking are considered to be one of the most frequent causes for revision after TKA without primary patellar resurfacing [15,16]. Approximately one third of patients revised in the first 5 years after TKA are attributed to instability or patellofemoral complications [15]. All except one of our patients with patellofemoral problems presented within the first five years after TKA. In a recent study Ahmad et al. suggested that bone scans could be helpful in patients with knee pain following TKA and they should be used as a screening tool in the diagnosis of patella-related problems [16]. SPECT/CT is superior to bone scanning in its ability to accurately localize the pathological tracer uptake within a small area of interest. Pagenstert et al. noted that the importance of SPECT/CT may be most pronounced in complicated anatomical sites such as the foot. This finding might also be true for the knee which consists of several different articular compartments (e.g. patellofemoral, medial and lateral tibiofemoral). The localization of the cause of pain is considered to be difficult [17]. With SPECT/CT we could differentiate between changes within the patellofemoral joint, which may be due to progression of patellofemoral OA, and other knee compartments, which may for example be due to mechanical loosening of the tibial or femoral prosthetic components. It was also feasible to analyse the tracer activity in the four different areas of the patella, by which correlations between metabolic changes and biomechanics could be drawn. This might lead to a better understanding of knee biomechanics.

Secondly, by characterisation of tracer uptake and accurate allocation to an anatomical area SPECT/CT clearly visualized the metabolic and patho-metabolic activity of the entire knee joint. The findings that patients with progression of patellofemoral OA or mechanical loosening of the femoral component showed significantly higher regional tracer uptake than others highlights the question whether we could establish a diagnostically relevant threshold and cut off value for tracer activity in patients with these problems. Along with these thresholds one could establish prognostically significant classifications indicating when a patient with knee pain after TKA should be revised.

Although Klett et al. [18] and others [19-21] described methods of quantitative analysis of SPECT images the direct comparability of these results is still limited as their defined ratios of tracer uptake are rather dependent on the reference regions chosen. In addition. the reproducibility of SPECT measurements in previous studies was only moderate in selected regions of interest [18-21].

An accurate definition and localization of the reference areas are needed but is still lacking. In our pilot series the femoral mid-shaft region was chosen as reference area. Ratios were calculated, but as their clinical value is questionable we decided to report the absolute values.

Another problem in the interpretation of SPECT tracer uptake is that increased tracer uptake may occur in 20% of patients within the first year after TKA even in asymptomatic knees with perfectly aligned TKA [11,22]. However, it is commonly agreed that diffusely intense uptake around the TKA is suspicious for loosening, infection, mechanical malalignment or progression of OA [22]. Clearly, no uptake around the TKA makes these causes of knee pain highly unlikely.

Generally the patella showed more tracer uptake than other zones, which is in accordance with Kantor et al. [22]. It might be explained by altered biomechanics after TKA, which in our series was evident in patients with tibial malrotation showing significantly increased uptake of the patella and the femur.

Patients with femoral mechanical loosening presented with increased tracer activity around the femoral tray which extended more frequently to the bone- prosthesis interface. This finding could be explained by micromotion of the prosthesis and subsequent stress on the periprosthetic bone resulting in osteoblastic activation [23]. Changes below the tibial tray are considered to be less specific [23].

Thirdly, measurement of prosthetic component position from the SPECT/CT data offered an additional benefit in patients with painful knee after TKA.

Malposition of prosthetic components is one of the most important factors leading to failure of TKA [15,24-28]. Chowdhury et al. [29] showed that external rotation of the tibial component of 15° caused posteromedial and anterolateral impingement. In addition, external tibial component rotation of 25° led to liftoff of the medial femoral condyle resulting in increased inlay stress due to point loading. With increased external rotation of the tibial component the contact of the patellofemoral joint shifted from medial towards the lateral facet. In contrast, this study showed that patients with a externally malrotated tibial tray had higher tracer activity in the medial patellar facet.

Within our pilot study we found that nearly half of the patients showed malrotation of the femoral or tibial component. The combination of SPECT and CT into one integrated system offers the conceptual advantage of correlating component position with tracer uptake in each anatomical area for patients.

However, the main weakness of our study was that there were too few patients for the study to have enough power to investigate the relationship of tracer uptake and component position in detail. Some differences did not reach statistical significance but might become significant once more patients are investigated.

Piloting our SPECT/CT algorithm on a limited consecutive cohort of patients with painful knees after primary TKA the composition of this group may not be representative of others. Hence, conclusions should be drawn with all due caution. However our study gives sufficient evidence to affirm that SPECT/CT has substantial clinical value in this subset of patients.

## Conclusions

On the basis of our results, we conclude that SPECT/CT is of great clinical value for the assessment and guidance of subsequent treatment in painful knees after TKA, particularly in patients with patellofemoral problems, malpositioned or loose components.

For further studies it would be extremely interesting to assess the question of clinical relevance of tracer uptake thresholds, to correlate component position with tracer uptake and to elucidate the natural course of tracer uptake before and after TKA.

## Competing interests

The authors declare that they have no competing interests.

## Authors' contributions

MH set up the protocol, organized ethics approval, carried out the study and drafted the manuscript. MH participated in the design of the study, the clinical and radiological follow-up and helped with the analysis of radiological data. PK and FI participated in the design of the study and helped with the data analysis. HR and AK helped with the data analysis and draft of the manuscript. NFF participated in the design of the study, interpretation of the results and helped with the draft of the manuscript. All authors read and approved the final manuscript.

## Pre-publication history

The pre-publication history for this paper can be accessed here:

http://www.biomedcentral.com/1471-2474/12/36/prepub
